# Supplementary material for: Central Regulation of Brown Fat Thermogenesis in Response to Saturated or Unsaturated Long-Chain Fatty Acids
Source: Int J Mol Sci. 2023 Jan 15;24(2):1697. doi: 10.3390/ijms24021697 (PMC9866012; doi:10.3390/ijms24021697)
Supplement: Supplementary file 1 [file ijms-24-01697-s001.zip › ijms-2150882-supplementary.pdf]

Supplementary information

Central regulation of brown fat thermogenesis in response to saturated or unsaturated long-chain fatty acids

Anna Fosch[1], Maria Rodriguez-Garcia[1], Cristina Miralpeix[2], Sebastián Zagmutt[1], Maite Larrañaga[1], Ana Cristina Reguera[1], Jesus Garcia-Chica[1], Laura Herrero[3,4], Dolors Serra[3,4], Nuria Casals[1,4] *, Rosalia Rodriguez-Rodriguez[1,4] *

[1] Basic Sciences Department, Faculty of Medicine and Health Sciences, Universitat Internacional de Catalunya, 08195 Sant Cugat del Vallès, Spain
[2] INSERM, Neurocentre Magendie, U1215, University of Bordeaux, 3300 Bordeaux, France
[3] Department of Biochemistry and Physiology, School of Pharmacy and Food Sciences, Institut de Biomedicina de la Universitat de Barcelona (IBUB), Universitat de Barcelona, Barcelona, 08028 Spain
[4] Centro de Investigación Biomédica en Red de Fisiopatología de la Obesidad y la Nutrición (CIBEROBN), Instituto de Salud Carlos III, 28029 Madrid, Spain

* Correspondence: rrodriguez@uic.es (R. R-R) and ncasals@uic.es (N.C.); Tel.: +34-935-042-002

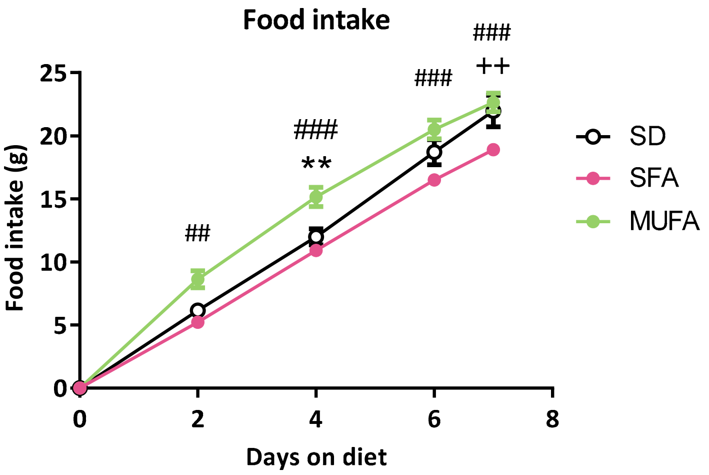

Supplementary Figure S1. Effects of short-term administration of SD, MUFA and SFA diets in food intake of WT mice after 7 days on special diets. Data were represented as mean ± SEM. **p<0.01 MUFA vs SD; ##p<0.01, ###p<0.001 MUFA vs SFA; ++p<0.01 SFA vs SD (n=7). MUFA: Diet with high content in monounsaturated fatty acids. SFA: Diet with high content in saturated fatty acids. SD: Standard diet.

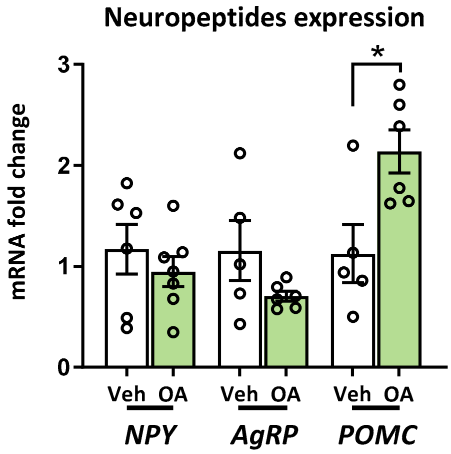

Supplementary Figure S2. Effects of oleic acid on hypothalamic levels of neuropeptides in WT mice. mRNA hypothalamic expression of the neuropeptides NPY, AgRP and POMC after oleic acid ICV administration. Data is represented as mean ± SEM, n= 6-7. *p<0.05. OA: Oleic acid; Veh: Vehicle.

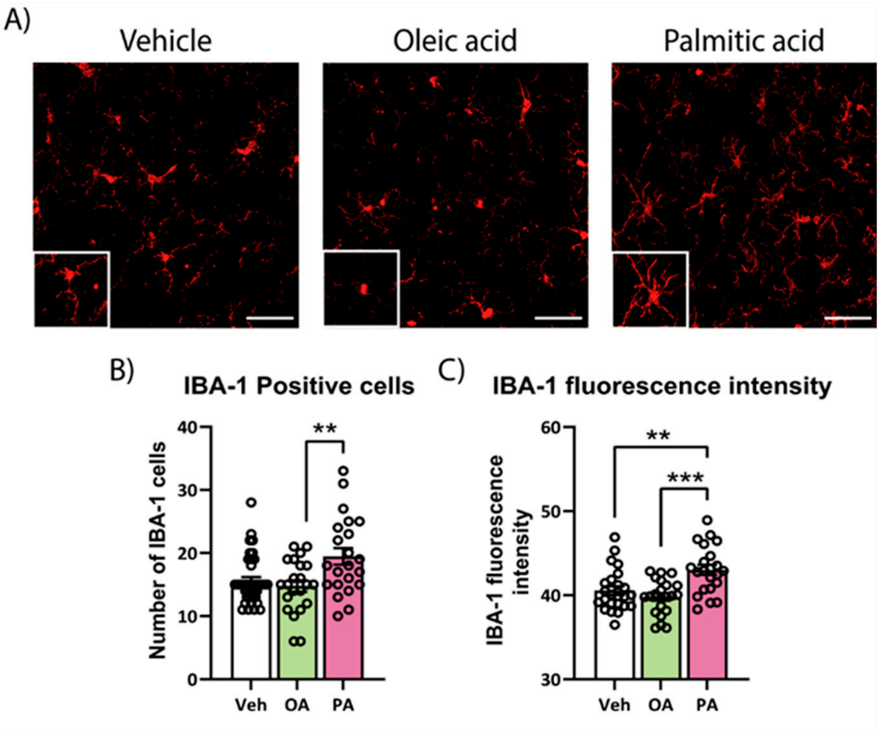

Supplementary Figure S3. Effects of oleic acid and palmitic acid on paraventricular inflammation in WT mice. A) Immunohistochemistry of IBA-1 expression after vehicle, oleic acid or palmitic acid administration in paraventricular hypothalamic nucleus (PaV). B) Quantification of IBA-1 positive cells per paraventricular nucleus. C) Quantification of fluorescence intensity per paraventricular nucleus. Data is represented as mean ± SEM (n=5, 5 slices/animal). **p<0.01, ***p<0,01 versus vehicle. A.U.: Arbitrary units. OA: Oleic acid; PA: Palmitic acid; Veh: Vehicle. Scale bar = 250μm.

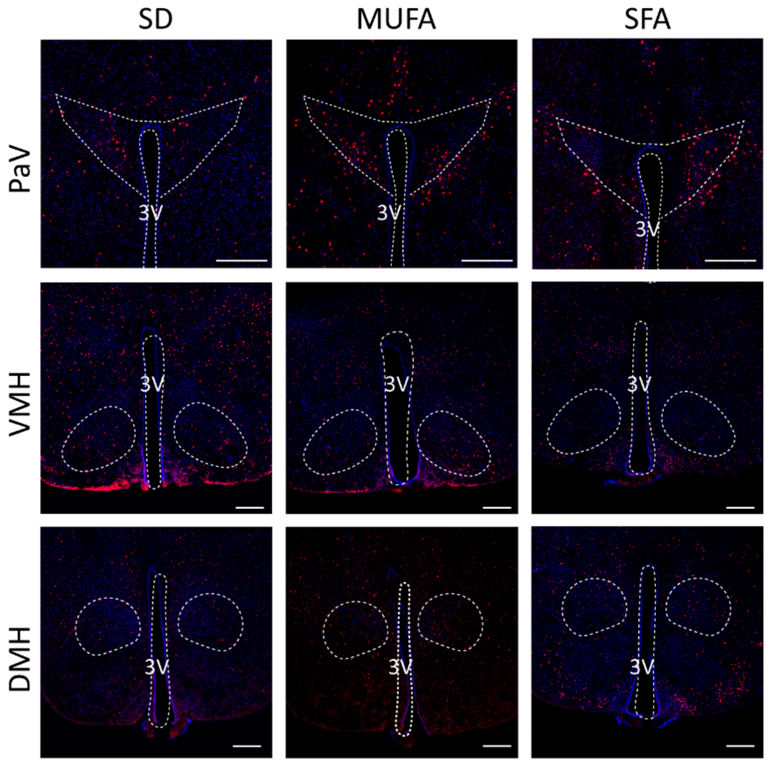

Supplementary Figure S4. Representative images for c-Fos expression (red) and nuclei staining with Hoechst (blue) in paraventricular (PaV), ventromedial (VMH) and dorsomedial hypothalamus (DMH) after 2 h exposition to SD, MUFA and SFA-enriched diets in WT mice. MUFA: diet with high content in monounsaturated fatty acids. SFA: diet with high content in saturated fatty acids. SD: Standard diet. 3V: third ventricle. Scale bar = 250 μm.

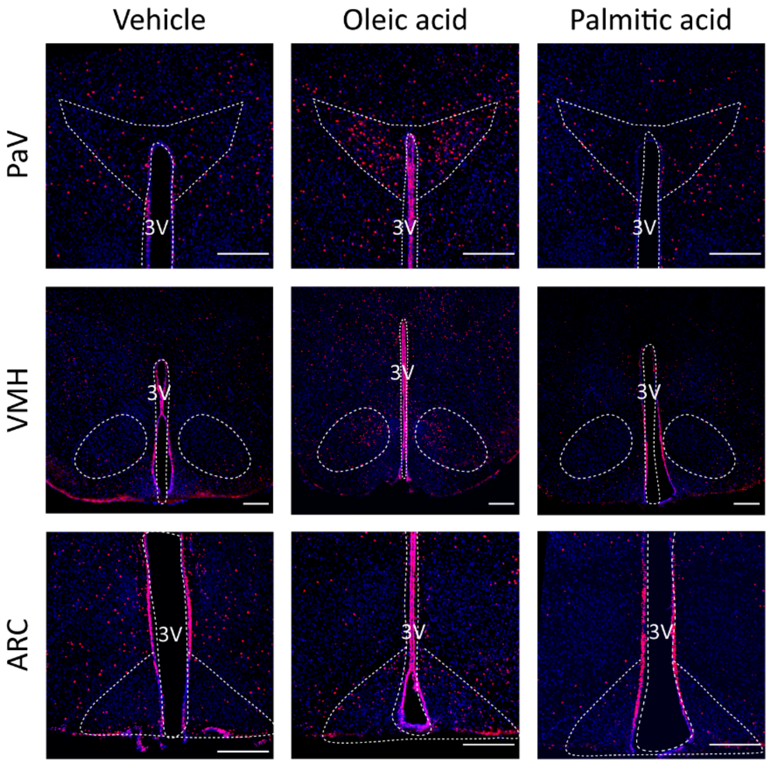

Supplementary Figure S5. Representative images for c-Fos expression (red) and nuclei staining with Hoechst (blue) in paraventricular (PaV), ventromedial (VMH) and arcuate nucleus of the hypothalamus (ARC) after 48 h of intracerebroventricular administration of fatty acids in WT mice. 3V: third ventricle. Scale bar = 250 µm.

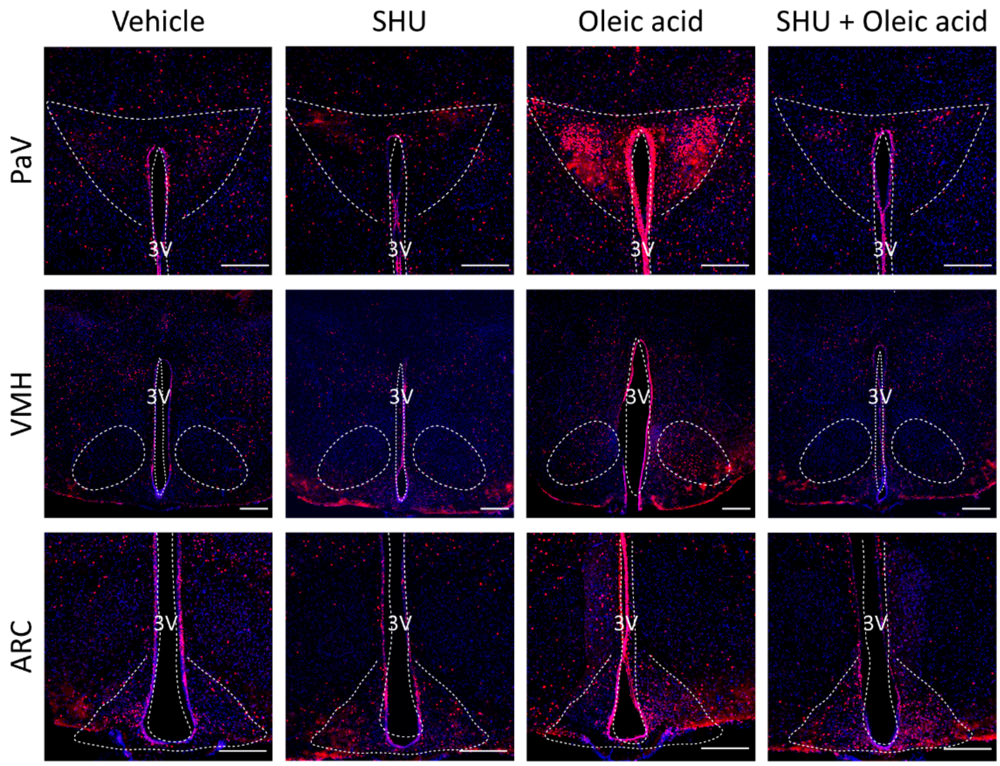

Supplementary Figure S6. Representative images for c-Fos expression (red) and nuclei staining with Hoechst (blue) in paraventricular (PaV), ventromedial (VMH) and arcuate nucleus of the hypothalamus (ARC) after 48 h of intracerebroventricular administration of oleic acid in the presence or absence of the MC4R antagonist SHU9119 (SHU) in WT mice. 3V: third ventricle. Scale bar = 250 μm.

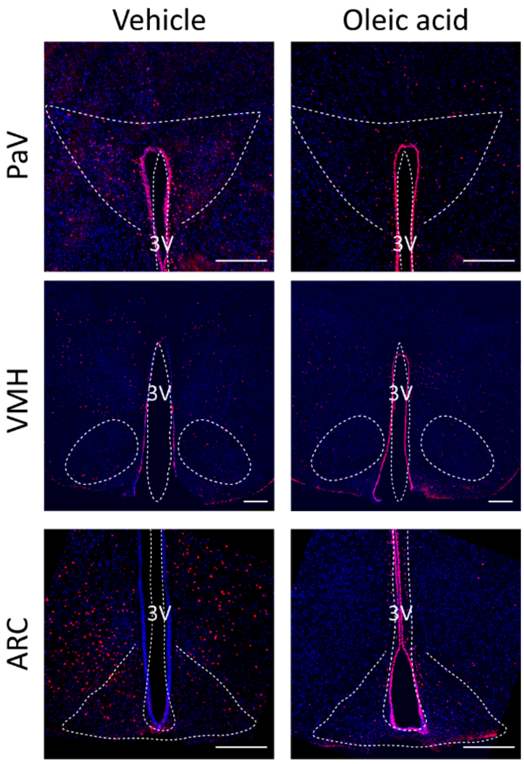

Supplementary Figure S7. Representative images for c-Fos expression (red) and nuclei staining with Hoechst (blue) in paraventricular (PaV), ventromedial (VMH) and arcuate nucleus of the hypothalamus (ARC) after 48 h of intracerebroventricular administration of oleic acid in CPT1C-KO mice. 3V: third ventricle. Scale bar = 250 µm.

Supplementary Table S1. List of the primers used for the RT-PCR assays.

**Gene**		**Sequence (5′ – 3′)**
AgRP	For	TTTGTCCTCTGAAGCTGTATGC
	Rev	GCATGAGGTGCCTCCCTA
Gapdh	For	TCCACTTTGCCACTGCA
	Rev	GAGACGGCCGCATCTTCTT
Npy	For	TCCGCTCTGCGACACTACAT
	Rev	TGCTTTCCTTCATTAAGAGGT
Pgc1α	For	GAAAGGGCCAAACAGAGAGA
	Rev	GTAAATCACACGGCGCTCTT
Pomc	For	TGAACATCTTTGTCCCCAGAG
	Rev	TGCAGAGGCAAACAAGATTGG
Prdm16	For	CCTAAGGTGTGCCCAGCA
	Rev	CACCTTCCGCTTTTCTACCC
Ucp1	For	CACACCTCCAGTCATTAAGCC
	Rev	CAAATCAGCTTTGCCTCACTC

Supplementary Table S2. Detailed information of the antibodies used for Western blotting and immunofluorescence assays.

**Antibody**	**Cat. number**	**Hybridization conditions**
Anti-ACC	3676 (Cell Signaling, Danvers, USA)	1:1000 (4ºC, o.n.)
Anti-pACC(Ser79)	3661 (Cell Signaling, Danvers, USA)	1:2000 (4ºC, o.n.)
Anti-AMPKα	23A3 (Cell Signaling, Danvers, USA)	1:1000 (4ºC, o.n.)
Anti-pAMPKα(Thr172)	2535S (Cell Signaling, Danvers, USA)	1:1000 (4ºC, o.n.)
Anti-FAS	sc-48357 (Santa Cruz, Dallas, USA)	1:1000 (4ºC, o.n.)
Anti-GAPDH	AM4300 (Ambion, Austin, USA)	1:50000 (4ºC, o.n.)
Anti-rabbit-HRP	111-035-144 (Jackson, West Grove, USA)	1:10000 (1h at r.t.)
Anti-mouse-HRP	515-035-003 (Jackson, West Grove, USA)	1:10000 (1h at r.t.)
Anti-c-Fos	2250 (Cell Signaling, Danvers, USA)	1:200 (1h at r.t.)
Anti-Iba1	019-19741 (Wako, Richmond, USA)	1:500 (1h at r.t.)
Anti-rabbit Alexa 647	A21244 (Invitrogen, Waltham, USA)	1:1000 (1h at r.t.)
